# Supplementary material for: Comprehensive Annotation and Functional Exploration of MicroRNAs in Lettuce
Source: Front Plant Sci. 2021 Dec 24;12:781836. doi: 10.3389/fpls.2021.781836 (PMC8739914; doi:10.3389/fpls.2021.781836)
Supplement: Supplementary file 3 [file Data_Sheet_9.ZIP › GSM2230753.plot/Lsa-miR397_Lsat_1_v5_gn_6_103081.1_1192_TPlot.pdf]

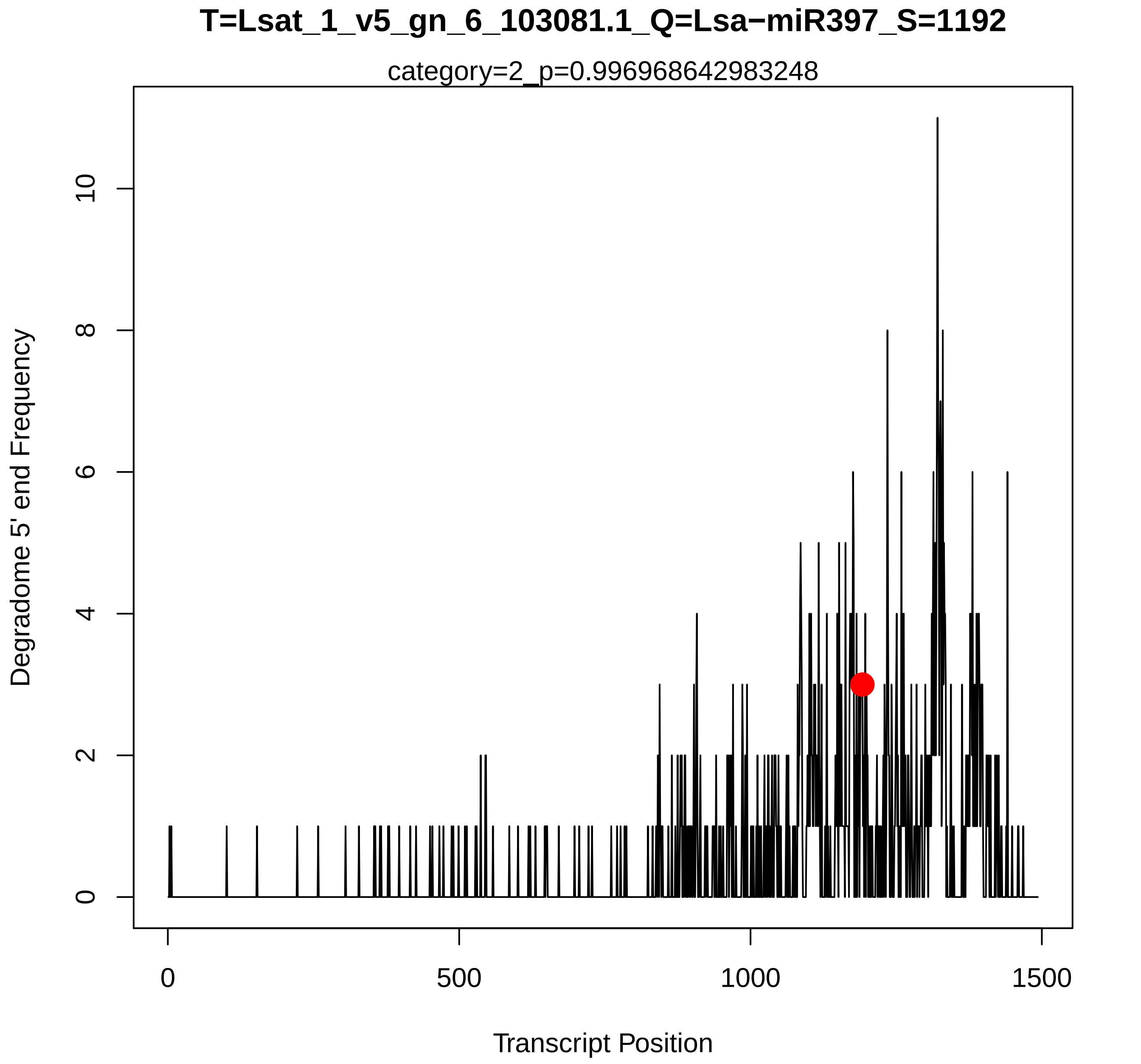

T=Lsat_1_v5_gn_6_103081.1_Q=Lsa−miR397_S=1192
category=2_p=0.996968642983248
Degradome 5' end Frequency
Transcript Position
0
2
4
6
8
10
0
500
1000
1500
